# Methylotrophic methanogenesis in the *Archaeoglobi* revealed by cultivation of *Ca.* Methanoglobus hypatiae from a Yellowstone hot spring

**DOI:** 10.1093/ismejo/wrae026

**Published:** 2024-03-07

**Authors:** Mackenzie M Lynes, Zackary J Jay, Anthony J Kohtz, Roland Hatzenpichler

**Affiliations:** Department of Chemistry and Biochemistry, Center for Biofilm Engineering, Thermal Biology Institute, Montana State University, Bozeman, MT 59717, United States; Department of Chemistry and Biochemistry, Center for Biofilm Engineering, Thermal Biology Institute, Montana State University, Bozeman, MT 59717, United States; Department of Chemistry and Biochemistry, Center for Biofilm Engineering, Thermal Biology Institute, Montana State University, Bozeman, MT 59717, United States; Department of Chemistry and Biochemistry, Center for Biofilm Engineering, Thermal Biology Institute, Montana State University, Bozeman, MT 59717, United States; Department of Microbiology and Cell Biology, Montana State University, Bozeman, MT 59717, United States

**Keywords:** archaea, MCR, methane, stable isotope tracing, thermophile, transcriptomics

## Abstract

Over the past decade, environmental metagenomics and polymerase chain reaction-based marker gene surveys have revealed that several lineages beyond just a few well-established groups within the *Euryarchaeota* superphylum harbor the genetic potential for methanogenesis. One of these groups are the *Archaeoglobi*, a class of thermophilic Euryarchaeota that have long been considered to live non-methanogenic lifestyles. Here, we enriched *Candidatus* Methanoglobus hypatiae, a methanogen affiliated with the family *Archaeoglobaceae*, from a hot spring in Yellowstone National Park. The enrichment is sediment-free, grows at 64–70°C and a pH of 7.8, and produces methane from mono-, di-, and tri-methylamine. *Ca*. M. hypatiae is represented by a 1.62 Mb metagenome-assembled genome with an estimated completeness of 100% and accounts for up to 67% of cells in the culture according to fluorescence *in situ* hybridization. Via genome-resolved metatranscriptomics and stable isotope tracing, we demonstrate that *Ca*. M. hypatiae expresses methylotrophic methanogenesis and energy-conserving pathways for reducing monomethylamine to methane. The detection of *Archaeoglobi* populations related to *Ca*. M. hypatiae in 36 geochemically diverse geothermal sites within Yellowstone National Park, as revealed through the examination of previously published gene amplicon datasets, implies a previously underestimated contribution to anaerobic carbon cycling in extreme ecosystems.

## Introduction

Methanogenesis is one of the most ancient metabolic pathways and plays a major role in the biogeochemical carbon cycle. Phylogenomic reconstructions and geological evidence suggest that methanogenesis was among the earliest metabolisms to evolve and that the last common ancestor of all extant archaea likely was a methanogen [1-9]. Therefore, the study of methanogens is essential for understanding the co-evolution of life and the biosphere. Methanogenic archaea are the primary producers of biogenic methane (CH_4_) and contribute ~60% to the estimated 576 Tg of annual global methane emissions to the atmosphere [10, 11]. Methanogenic pathways are classified by their carbon and electron sources [12-14]. All methanogenic pathways converge at the terminal methane-forming step catalyzed by the methyl-coenzyme M reductase (MCR) complex. MCR and its homologs also catalyze the reverse reaction in the anaerobic oxidation of alkanes in alkanotrophic archaea [15, 16]. MCR is uniquely present in all methanogens and is commonly used to identify potential methane and/or alkane cycling archaea in sequencing surveys [12, 17].

The physiology and biochemistry of methanogens have near-exclusively been investigated in axenic cultures of microorganisms belonging to the *Euryarchaeota* superphylum [12, 17-19]. These predominantly grow by acetoclastic or CO_2_-reducing hydrogenotrophic methanogenesis, with only rare observations of *Euryarchaeotal* methyl-reducing methanogens [12, 20, 21]. As a result, despite the dominance of methyl-based methanogenesis in anoxic environments with high salt and/or high sulfate concentration (e.g. anoxic marine sediments, coastal wetlands, hypersaline lakes), methylotrophic methanogenesis has in the past often been considered to be of comparatively limited environmental distribution. The extensive use of environmental metagenomics has led to the discovery of metagenome-assembled genomes (MAGs) encoding MCR from new lineages that are prevalent in anoxic environments, both within and outside the *Euryarchaeota* [2, 12, 22-26].

The majority of MAGs affiliated with archaeal phyla outside the *Euryarchaeota* are predicted to be methyl-reducing methanogens, with the exception of *Candidatus* (Ca.) Nezhaarchaeota [25, 27] and *Ca*. Methanomixophus affiliated with the order *Archaeoglobales*, which have been hypothesized to be CO_2_-reducing hydrogenotrophic methanogens [12, 25, 28]. This result is consistent with the observation that methylated methanogenic substrates, including methylamines and methanol, are prevalent in the environment, although their concentrations in hot springs is currently unknown. Furthermore, methyl-reducing methanogenesis is considered the predominant mode of methanogenesis in anoxic marine, freshwater, and hypersaline sediments (reviewed in Bueno de Mesquita *et al.* [20]).

Members of the class *Archaeoglobi* have long been considered non-methanogenic with isolates characterized as dissimilatory sulfate reducers brought into culture as early as 1987 [29]. To date, only nine species of the class *Archaeoglobi* have been obtained in axenic culture, and all were sourced from marine hydrothermal systems or off-shore oil reservoirs [30]. The discovery of both MCR [25, 31, 32] and methyl-H_4_M(S)PT:coenzyme M methyltransferase (MTR) complexes in genomes of the *Archaeoglobaceae* has suggested that members of this family may live by methanogenesis [28].

Very recently, important progress toward experimental verification of methanogenesis by members of this family has been made. Liu *et al*. reported the *in situ* expression of genes related to hydrogen-dependent methylotrophic methanogenesis and heterotrophic fermentation within populations of *Archaeoglobi* in a high-temperature oil reservoir [28]. Lynes *et al*. reported that *Archaeoglobi* can be enriched in hot spring mesocosms under methanogenic conditions [33]. Wang *et al*. reported that *mcrABG* and other methanogenesis marker genes encoded by two *Archaeoglobales* MAGs were highly expressed in hot spring microcosms incubated at 65°C and 75°C [34]. Importantly, one of these *Archaeoglobales* MAGs represented the only Mcr-encoding archaeon that expressed *mcrABG* genes in methanogenic microcosms performed without substrate addition or with the addition of 10 mM methanol at 75°C. This indirectly demonstrated the methanogenic nature of this archaeon [34]. Last, Buessecker *et al*. reported the establishment of a methanogenic enrichment culture of *Ca*. Methanoglobus nevadensis from Great Boiling Spring (GBS) (NV, USA) [35]. The culture yields up to 158 μM methane after 2 weeks of incubation at its optimal growth temperature of 75°C. *Ca. M. nevadensis* is represented by a 63% complete MAG obtained from the culture and a 98% complete MAG obtained a decade earlier [35].

Here, we report on the enrichment cultivation of *Ca*. Methanoglobus hypatiae LCB24, a methanogen affiliated with the family *Archaeoglobaceae*, from a hot spring in Yellowstone National Park (YNP). Using a combination of targeted cultivation, growth experiments, microscopy, stable isotope tracing, metagenomics, and metatranscriptomics, we demonstrate that *Ca*. M. hypatiae lives by methylotrophic methanogenesis and converts different methylamines to methane. By examining previously published datasets for the presence of Mcr-encoding *Archaeoglobi*, we demonstrate that these archaea are distributed in geothermal features of YNP, where they likely contribute to anaerobic carbon cycling. Our study presents direct evidence of methanogenesis within the *Archaeoglobaceae* and adds to the growing body of evidence demonstrating that methanogenesis is widely spread within the *Euryarchaeota* superphylum.

## Materials and Methods

All chemicals used in this study were sourced from Sigma Aldrich unless otherwise specified.

### Sample collection, enrichment, and cultivation

In November 2021, a slurry of sediment and water (1:9) was collected from an unnamed hot spring in the Lower Culex Basin of YNP, WY, USA. In our previous survey of Mcr-encoding archaea in YNP [33], this hot spring was given the identifier LCB024 (44.573294, −110.795388; pH 7.8, 67°C). A mixture of surface sediment (~1–2 cm deep) and hot spring water was collected into a glass bottle and sealed headspace-free with a thick butyl rubber stopper. Collected material was transported back to the lab and stored at room temperature. Using this material as inoculum, 30 ml enrichments were established in February 2022 in 60 ml serum bottles. Material was homogenized by mixing and was then diluted 1:10 (v/v) with anoxic medium in an anoxic glove box (N_2_/CO_2_/H_2_; 90/5/5%).

Medium was prepared anoxically as described previously [36]. Basal mineral medium contained a base of (per liter): KH_2_PO_4_, 0.5 g; MgSO_4_·7H_2_O, 0.4 g; NaCl, 0.5 g; NH_4_Cl, 0.4 g; CaCl_2_·2H_2_O, 0.05 g; HEPES, 2.38 g; yeast extract, 0.1 g; and 0.002% (w/v) (NH_4_)_2_Fe(SO_4_)_2_·6H_2_O. Medium was transferred to a Duran flask with a side opening and autoclaved for 20 m at 121°C*.* Medium was then further supplemented with 5 mM NaHCO_3_, 1 ml trace element solution SL-10, 1 ml Selenite-Tungstate solution, 1 ml CCM vitamins [37], 0.0005% (w/v) resazurin, 10 mg of coenzyme-M, 2 mg sodium dithionite, 1 mM dithiothreitol, 1 mM Na_2_S·9H_2_O, with pH adjusted to 7.8 using sodium hydroxide (NaOH, 12 N). Serum bottles were sealed with butyl rubber stoppers and aluminum crimps before the headspace were exchanged with N_2_ (99.999%) for 5 min and set to a 200 kPa N_2_ atmosphere. Monomethylamine (MMA) was added from a filter-sterilized methylamine-hydrochloride anoxic stock solution to a final concentration of 10 mM. The bacterial antibiotics streptomycin (50 mg/L; inhibitor of protein synthesis) and vancomycin (50 mg/L; inhibitor of peptidoglycan synthesis) were added from filter-sterilized anoxic stock solutions. The enrichments were incubated at 70°C in the dark without shaking. Cultures were maintained by regular transfer of 10% v/v into fresh media, which contained MMA and antibiotics. A sediment-free culture was obtained after the third transfer after which it was transferred at 10% v/v to 50 ml in 125 ml serum bottles.

### Stable isotope tracing

The conversion of ^13^C- or D_3_-MMA (^13^CH_3_-NH_2_, CD_3_-NH_2_) to ^13^CH_4_ or CD_3_H was tracked by incubating active enrichment cultures in the presence of 20% labeled substrate (98%; Cambridge Isotope Laboratories). Incubations were carried out in 30 ml culture volumes in 60 ml serum bottles with 8% v/v inoculum, 50 mg/L streptomycin, 50 mg/L vancomycin, 10 mM MMA, and N_2_ gas (99.999%) incubated in anoxic media (pH 7.8, 70°C) in six replicates (SI Appendix, Fig. S3). Duplicate control incubations included (i) ^12^C-MMA and (ii) inoculum without MMA. Triplicate control incubations were performed with (iii) ^12^C-MMA plus 10 mM bromoethanesulfonate (BES) added in mid-exponential phase (Day 33) to inhibit methanogenesis and (iv) 10 mM BES added at time of inoculation (Day 0) without substrate. Headspace samples were collected throughout the experiment as described above and analyzed using a Shimadzu QP2020 NX GCMS equipped with a GS-CarbonPLOT column (30 m × 0.35 mm; 1.5 μm film thickness; Agilent) and operated in Selected Ion Monitoring mode. The instrument was operated using the method described in Ai *et al*. [38] with helium as a carrier gas. All injections were performed by a Shimadzu AOC-6000 autosampler robot. Peak areas corresponding to m/z ratios of 16 for ^12^CH_4_, 17 for ^13^CH_4_, and 19 for CD_3_H were used for quantification.

### Metagenomic sequencing, assembly, and annotation

**Figure 1:**
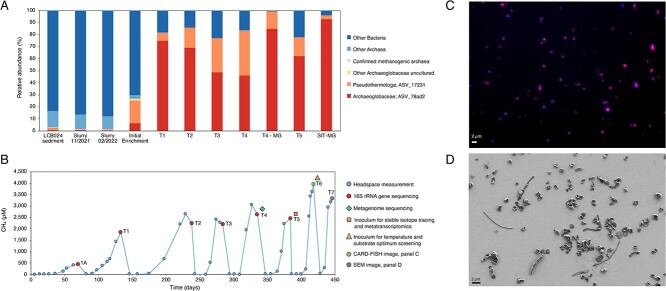
Community composition and methane production of the methanogenic enrichment culture containing *Ca*. M. hypatiae LCB24; (A) relative abundance of 16S rRNA gene amplicons in the initial sediment from hot spring LCB024, the slurry collected in November 2021, slurry material used to initiate enrichments in February 2022, the initial enrichment, and five subsequent transfers (T1–T5) are shown; for comparison, the estimated relative abundance of two metagenomic samples (T4-MG and SIT-MG) is included; the metagenome recovered from a replicate from the stable isotope tracing experiment incubated in the presence of deuterated methylamine (SIT-MG) revealed *Ca*. M. hypatiae grew to 92.8% relative abundance during the experiment; the two most abundant ASVs across enrichment transfers are shown with other taxa collapsed; other methanogenic archaea were not identified in the initial enrichment or in any subsequent transfer; relative sequence abundance for all ASVs is reported in SI Appendix, Table S1; (B) headspace methane produced over long-term cultivation; the time between transfers decreased while the average maximum concentration of methane increased over time; culture 1A represents the initial enrichment; a history of methane measurements can be found in SI Appendix, Table S2; (C) visualization of *Ca*. M. hypatiae cells at T6 labeled via CARD-FISH by the general archaea probe Arch915 (red). DAPI staining of cells is in blue; (D) cell morphologies in enrichment culture LCB24 at T7 as observed by SEM.

Two metagenomes were obtained over the course of this study. A 42 ml aliquot of the fourth transfer of the enrichment (Fig. 1 T4-MG) was filtered onto a 0.22 μm filter. The filter was transferred to a lysing matrix E tube and DNA extracted immediately following filtration. Genomic DNA was extracted using the FastDNA Spin Kit for Soil (MP Biomedicals, Irvine, CA) following the manufacturer’s guidelines.

A second metagenome was recovered from one of the six culture replicates grown in the presence of CD_3_-NH_2_ and used for recruiting transcriptomic reads from the other replicates (Fig. 1 SIT-MG). A 60 ml syringe flushed with N_2_ gas was used to transfer 30 ml of culture to a sterilized oak ridge tube. Cells were harvested through centrifugation for 30 min at 10000 rpm at 4°C. The supernatant was removed, and DNA extracted from the pellet using the FastDNA Spin Kit for Soil (MP Biomedicals, Irvine, CA) following the manufacturer’s guidelines. Genomic DNA for both metagenomes was shipped to SeqCenter (Pittsburgh, PA), and sample libraries were prepared using the Illumina DNA Prep kit and 10 bp unique dual indices (UDIs). The first metagenome (T4-MG) was sequenced on a NextSeq 2000 System (Illumina) and the second (SIT-MG) sequenced on a NovaSeq 6000 System (Illumina), each producing 2 × 151 bp reads. Demultiplexing, quality control, and adapter trimming were performed with bcl-convert v3.9.3. The quality of the reads was evaluated with FastQC before quality, linker and adapter trimming, artifact and common contaminant removal, and error correction were performed with the rqcfilter2 pipeline (maxn = 3, maq = 10, trimq = 20) and bbcms (mincount = 2, hcf = 0.6). Resulting reads were assembled with SPAdes v3.15.13 (Nurk, 2017) (−k 33,55,77,99 127 —meta –only-assembler), and coverage was determined with bbmap v38.94 (ambiguous = random) (https://sourceforge.net/projects/bbmap) [39]. In addition to the initial assembly, co-assemblies using both T4-MG and SIT-MG metagenomes were also performed [1] with reads directly fed into SPAdes with the—only-assembler option excluded; and [2] with the trimmed and error corrected reads and the same SPAdes parameters as above. The statistics of MAGs generated through various assembly and quality control methods were evaluated, and the approach that produced the highest quality MAG was chosen for subsequent analysis (Dataset S1). The quality was determined by considering factors such as the number of resulting sequences, total length, completeness, and the minimum, maximum, and average sequence lengths. Annotation of the assembled sequences was performed with Prokka v1.14.16 [40]. Assembled scaffolds ≥2000 bp were binned using Maxbin v2.2.7 [41], Metabat v2.12.1 (with and without coverage) [42], Concoct v1.0.0 [43], Autometa v1 (bacterial and archaeal modes with the machine learning step) [44], followed by bin refinement with DAS_Tool v1.1.6 [45], as previously described [46]. Bin completeness and redundancy were assessed with CheckM v1.2.2 [47].

### RNA extraction, sequencing, and transcriptomic processing

Total RNA was extracted for transcriptomics from four of the six replicates of *Archaeoglobus* cultivated in the presence of labeled substrate (^13^CH_3_-NH_2_ or CD_3_-NH_2_) for a total of eight replicates. Each replicate culture in the exponential growth phase (Day 32) was moved from the 70°C incubator to an ice bath placed at −20°C for 40 mins to stop cellular activity. A 60 ml syringe flushed with N_2_ gas was used to transfer 30 ml of culture to a sterilized oak ridge tube and kept on ice. Cells were harvested through centrifugation for 30 min at 10000 rpm at 4°C. The supernatant was removed, and the pellet transferred to a lysing matrix E tube (MP Biomedicals, Irvine, CA) to which 600 μL of RNA lysis buffer was added. Samples were homogenized in a MP Bioscience FastPrep instrument for 40 s at a speed setting of 6.0 m/s followed by centrifugation for 15 min at 14000 rpm. RNA was extracted using the Quick-RNA miniprep kit (Zymo Research, Irvine, CA) including a DNAse treatment step and eluted in 50 μL of RNAse free water. Centrifugation steps were performed at 15000 rpm and the final spin for elution at 10000 rpm. Of the eight replicates extracted, six measured >50 ng/μL (3× ^13^CH_3_-NH_2_ and 3× CD_3_-NH_2_) and were sent for transcriptomic sequencing at SeqCenter (Pittsburgh, PA). Samples were DNAse treated with Invitrogen DNAse (RNAse free). Library preparation was performed using Illumina’s Stranded Total RNA Prep Ligation with Ribo-Zero Plus kit and 10 bp UDI. Sequencing was done on a NovaSeq 6000, producing paired end 151 bp reads. Demultiplexing, quality control, and adapter trimming were performed with bcl-convert (v4.1.5). Read quality was further evaluated with FastQC v0.11.9 [48] before quality trimming and artifact, rRNA, and common contaminant removal with the rqcfilter2 pipeline (trimq = 28, maxns = 3, maq = 20), and error correction with bbcms (mincount = 2, hcf = 0.6) from the BBTools suite v38.94 [39]. Additional rRNA gene reads were detected and removed with Ribodetector v0.2.7 [49], and any remaining rRNA gene reads were finally removed with bbmap, using rRNA genes recovered from the metagenomes (see below) as references. The resulting mRNA reads were mapped against annotated genes from the paired metagenomes with bbmap to calculate reads per kilobase of transcript per million mapped read (RPKM) (ambig = random).

## Results and Discussion

### Cultivation

In our recent survey on the diversity of Mcr-encoding archaea in the geothermal features of YNP, mesocosms seeded with biomass from a hot spring located within the Lower Culex Basin (LCB024; pH 7–8, 56–74°C) had shown potential to enrich for methanogenic *Archaeoglobi* [33]. Using a sediment slurry collected from LCB024, we initiated incubations supplied with MMA and antibiotics incubated in anoxic media (pH 7.8, 70°C) under a N_2_ headspace. The relative abundance, as determined by 16S rRNA gene amplicon sequencing, of *Archaeoglobi*-affiliated organisms in LCB024 was 0.32% in the initial slurry and had fallen to 0.02% by the time incubations were initiated a few months after samples had been collected (Fig. 1A).

Methane was detected after 36 days in the initial enrichment, and the culture transferred to fresh media after reaching the late exponential phase of methane production following 70 days of incubation (447 μM; Fig. 1B). Five *Archaeoglobi*-related 16S rRNA gene amplicon ASVs were identified in the initial enrichment; however, one ASV grew to dominate the microbial community after the first transfer and reached 74.8% relative abundance after 62 days. In the transfers that followed, *Archaeoglobi*-related sequences became the only archaeal reads detected by 16S rRNA gene amplicon sequencing with the second most abundant organism a bacterium affiliated with the *Pseudothermotoga* at 6.8%. Although the CO_2_-reducing methanogen *Methanothermobacter* sp. was detected at 0.45% relative abundance in the slurry material used for inoculation, it was not detected in any subsequent transfers, nor were any known methanogens. Over subsequent transfers (238 days, T2–T5), the relative abundance of *Archaeoglobi* ASVs ranged from 46% to 69%, and the final methane yield steadily increased from 1844 to 2459 μM. A sediment-free enrichment was obtained by the third transfer. Starting with the fourth transfer, the culture volume was scaled from 30 ml to 50 ml. By the sixth transfer, the culture produced 3943 μM methane within 34 days. Metagenomic sequencing at two timepoints (Day 335 of the enrichment and Day 33 of the isotope tracing experiment described below) and 16S rRNA gene amplicon sequencing over recurring transfers (Fig. 1A) demonstrated that ASVs and MAGs affiliated with *Archaeoglobi* represented the only archaeon in culture LCB24. A single MCR complex (*mcrAGCDB*) belonging to the *Archaeoglobi* MAG was present, indicating this MAG represents the only methanogenic population.

### Metagenomics and phylogenetics

**Figure 2:**
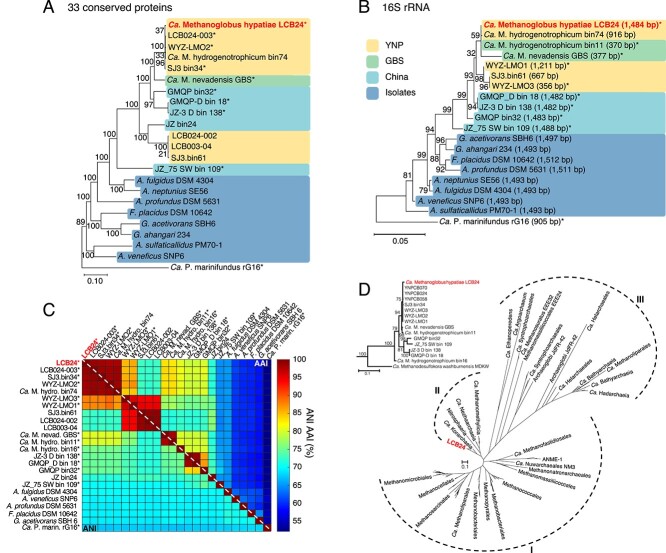
Phylogenetic affiliation of *Ca*. M. hypatiae LCB24; (A) maximum-likelihood tree, inferred with fasttree and WAG model (midpoint root), using a concatenated alignment of 33 conserved single copy proteins (list provided in SI Appendix, Table S4); references are colored by the habitat or type from which sequences had been recovered: hot springs in YNP, yellow; GBS, green; hot springs in China, blue; isolates from marine hydrothermal vent systems, dark blue; (B) maximum-likelihood tree inferred with fasttree using 16S rRNA genes with length in base pairs (bp); (C) ANI and AAI analysis of reference *Archaeoglobales* MAGs and genomes; asterisks (*) indicate MAGs containing *mcrA*, apart from the MAG of *Ca*. M. hydrogenotrophicum bin74 which encodes a *mcrA* that is interrupted by a stop codon; AAI and ANI values are provided in SI Appendix, Fig. S1; (D) maximum-likelihood tree, inferred with IQtree2 and the LG + C60 + F + G model, from the amino acid alignment of McrA; dashed lines indicate McrA/AcrA groups: (I) McrA from methanogens and ANME (MCR-type), (II) McrA from TACK lineages (MCR-type), (III) McrA-like from proposed and experimentally confirmed alkane oxidizing archaea (ACR-type); insert shows MAGs closely related to *Ca*. M. hypatiae LCB24.

The reconstructed Mcr-encoding *Archaeoglobi* MAG from culture LCB24 was 1.62 Mbp in length with an estimated completeness of 100% according to checkM (SI Appendix, Table S3). This MAG was the result of a combined assembly of the T4-MG and SIT-MG metagenomes as this method yielded an improved assembly. Therefore, it was used for phylogenomic analysis against *Archaeoglobi* reference MAGs and genomes using 33 conserved single copy marker proteins and 16S rRNA genes (Fig. 2A and B, SI Appendix, Table S4). The phylogenomic analysis showed that MAGs encoding MCR complexes clustered separately from those lacking *mcr* gene sequences. Consistently, 16S rRNA gene phylogeny supported this clustering with a pronounced separation of hot spring reference genomes and MAGs from current known isolates of *Archaeoglobi*, resulting in three main clusters: (i) those retrieved from North American hot springs (YNP and GBS), (ii) those originating from hot springs in China, and (iii) isolates, all of which were obtained from deep-sea marine hydrothermal systems (Fig. 2B).

LCB24 and closely related reference MAGs and isolate genomes exhibited a range of amino acid identities (AAI, 52.6%–98.6%; Fig. 2C). Altogether, the LCB24 MAG was found to be highly related to previously obtained *Archaeoglobi* MAGs encoding the MCR complex and only distantly related to other *Archaeoglobales* sp. (AAI, 58.9%–65%; average nucleotide identity (ANI), 70.3%–70.6%; 16S rRNA ANI, 91.6%–93.8%; SI AppendixFigs S1 and S2). Based on AAI, MAG LCB24 was most closely related to *Archaeoglobi* LCB024-003 MAG (AAI, 98.6%), which we had obtained from the same hot spring in a previous study [33]. The ANI and AAI values to the closest cultured methanogen, *Ca*. Methanoglobus nevadensis GBS, are 80.2% and 83.3%, respectively. Based on these results, we designate this archaeon *Ca*. M. hypatiae strain LCB24, named after the philosopher Hypatia of Alexandria (for a protologue, see the SI Appendix, Results and Discussion). The estimated relative abundance of *Ca*. M. hypatiae based on the SIT-MG was 92.8%. Other community members in the LCB24 culture with >1% relative sequence abundance included members of the *Pseudothermotoga* (3.2%), *Desulfovirgula* (1.7%), and the family *Moorellaceae* (1.3%) (Fig. 1A, Dataset S1).

The only *mcrAGCDB* genes recovered from both metagenomes belong to the genome of *Ca*. M. hypatiae. Phylogenetic analysis of the single copy of McrA indicated its close relationship to McrA sequences found in members of the TACK superphylum (Fig. 2D). This contrasts with the placement of *Ca*. M. hypatiae within the *Euryarchaeota* based on phylogenomics (Fig. 2A), suggesting that *Archaeoglobi* could have obtained the MCR complex as a result of a horizontal gene transfer event from an archaeon in the TACK superphylum [7, 8]. Also, it could indicate that non-methanogenic *Archaeoglobi* lost the capacity for anaerobic methane cycling after they had diverged from a shared methanogenic ancestor.

### Methanogenic activity of *Ca*. M. hypatiae

**Figure 3:**
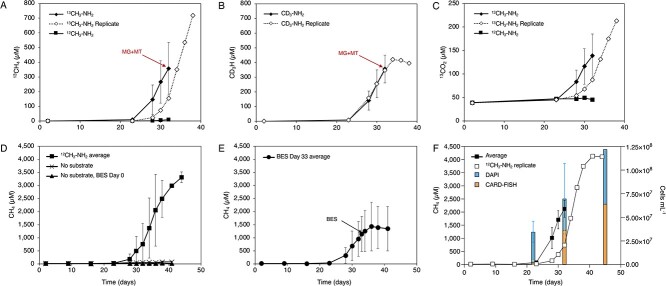
Conversion of stable isotope labeled MMA to methane by culture LCB24; (A) production of ^13^CH_4_ in cultures amended with ^13^CH_3_-NH_2_ vs. ^12^CH_3_-NH_2_ (six replicates); (B) production of CD_3_H in cultures amended with CD_3_-NH_2_ (six replicates); (C) production of ^13^CO_2_ in cultures amended with ^13^CH_3_-NH_2_ vs. ^12^CH_3_-NH_2_; for plots A and B, 10 total replicates across treatments were sacrificed during mid-exponential phase for metagenomic or metatranscriptomic sequencing indicated by red arrows; ^13^CH_4_, CD_3_H, or ^13^CO_2_ production for the replicate allowed to reach stationary phase is shown as a dashed line through open diamond symbols; (D) production of ^12^CH_4_ in cultures amended with ^12^C-MMA (^12^CH_3_-NH_2_; 2 replicates); cultures incubated without substrates (two replicates) and those to which the inhibitor BES was added on Day 0 (3 replicates) did not produce ^12^CH_4_ over the course of the experiment; (E) production of ^12^CH_4_ in cultures amended with ^12^CH_3_-NH_2_ to which BES was added on Day 33 of incubation (black arrow; three replicates); the average production of ^12^CH_4_ leveled off and ceased after the introduction of BES, indicating methane generation by *Ca*. M. hypatiae is MCR-dependent; error bars indicate standard deviation of biological replicates when applicable; measurements of ^12^CH_4_, ^13^CH_4_, CD_3_H, and ^13^CO_2_ for all replicates and controls are reported in Dataset S2; ^12^CH_4_ measurements for all controls and replicates are shown in SI Appendix, Fig. S4 and Dataset S3; (F) ^12^CH_4_ production and fraction of *Ca*. M. hypatiae cells in biological replicates incubated with ^13^CH_3_-NH_2_; relative abundance of cells was determined at three time points (Days 22, 32, 45) based on the fraction of *Ca*. M. hypatiae specific CARD-FISH counts (orange) versus total counts of DAPI-stained cells (blue); error bars indicate the standard deviation for four biological replicates on Days 22 and 32.

To gain insight into the activity of *Ca*. M. hypatiae under methanogenic and non-methanogenic conditions, a stable isotope tracing (SIT) experiment was conducted. Cultures were incubated in the presence of 10 mM of MMA; 8 mM of substrate were isotopically light, whereas the remaining 2 mM consisted of either ^13^C-MMA (^13^CH_3_-NH_2_) or D_3_-MMA (CD_3_-NH_2_). Addition of the methanogenesis inhibitor BES was used as a non-methanogenic control (Figs 1B and 3, SI Appendix, Fig. S3). On average across six replicates, the cultured converted ^13^CH_3_-NH_2_ to 356 μM ^13^CH_4_ (17.8%) and 138.71 μM ^13^CO_2_ (6.9%) by Day 32 (Fig. 3A and C, Dataset S2). The conversion of CD_3_-NH_2_ was nearly identical yielding 355 μM CD_3_H (Fig. 3B). In the exponential phase of methane production, five of the six replicates were harvested for metagenomic and metatranscriptomic sequencing, while the sixth replicate was allowed to grow to stationary phase. The replicate allowed to grow in each respective experiment converted the provided ^13^CH_3_-NH_2_ to 717.7 μM ^13^CH_4_ (35.9%) and 212.95 μM ^13^CO_2_ (10.65%) or CD_3_-NH_2_ to 394.76 μM CD_3_H (19.7%) by Day 38 (Fig. 3A–C). These results confirmed that MMA was converted to methane by the LCB24 culture. The production of ^13^CO_2_ may represent the dismutation of ^13^CH_3_-NH_2_ to generate the reducing power for methanogenesis via the methyl-branch of the Wood–Ljungdahl pathway (WLP) or may be explained by other organisms in the culture catabolizing MMA. Yet, no transcriptomic evidence for this activity was present in this experiment. No methane production was observed for cultures treated with BES or in cultures incubated without MMA (Fig. 3D). When BES was added to cultures in the exponential phase, methane production ceased indicating the generation of methane is reliant on the *Archaeoglobi* MCR (Fig. 3E).

### Visualization and cell enumeration

The growth of *Ca*. M. hypatiae was tracked in four replicates during the SIT experiment with catalyzed reporter deposition fluorescence *in situ* hybridization (CARD-FISH) using a general archaea-targeted probe Arch915 [50] and DNA-staining (DAPI) (Fig. 1C). As the production of methane increased throughout the experiment, there was a concurrent rise in the relative cell abundance of *Ca*. M. hypatiae (Fig. 3F, SI Appendix, Table S5). The initial assessment on Day 22 across four replicates revealed the total cell density to be 3.45 × 10^7^ ± 1.14 × 10^7^ before substantial concentrations of methane had been detected in the headspace (<132 μM). By Day 32, methane concentrations reached 1777 ± 739 μM and the total cell density increased to 6.97 × 10^7^ ± 3.73 × 10^7^ cells ml^−1^ with 54% (±9.6%) of cells labeled as *Ca*. M. hypatiae (Fig. 3F). All but one of these replicates were then sacrificed for further analysis. Finally on Day 45, the remaining replicate reached a headspace methane concentration of 4109 μM and a total cell density of 1.22 × 10^8^ with 53% of cells labeled as *Ca*. M. hypatiae.

Visualization of the enrichment culture via scanning electron microscopy (SEM) revealed that most cells exhibited a regular to irregular coccoid morphology, with a width ranging from 0.5 to 1 μm (Fig. 1D). This morphology has previously been described for other *Archaeoglobi* species [30, 51-53].

### Alternative substrates and temperature optimum

**Figure 4:**
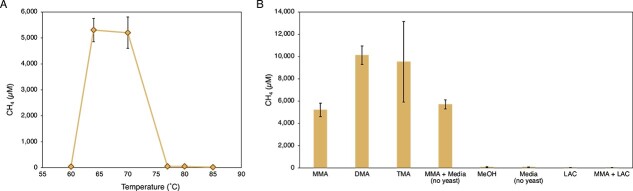
Temperature and substrate range of culture LCB24; (A) methane production from MMA was observed between 64 and 70°C; (B) substrate range; methane production was observed for MMA, DMA, TMA, and in media prepared without yeast extract; MeOH, methanol; both experiments performed in triplicate; all measurements can be found in Dataset S4.

We determined the substrate and temperature range of *Ca*. M. hypatiae by growing the culture in the presence of several substrates at 70°C or with 10 mM MMA at 60–85°C (Fig. 4A and B). Conditions that lead to the production of methane included 10 mM trimethylamine (TMA), 10 mM dimethylamine (DMA), 10 mM MMA in media without yeast extract, and the control with 10 mM MMA and 0.01% yeast extract. Methane production of cultures grown with MMA in the presence or absence of yeast extract was indistinguishable (5202 ± 606 and 5703 ± 410 μM CH_4_, respectively) indicating that yeast extract is not essential for methanogenic growth. Observed methane concentrations were higher in incubations amended with DMA (10 115 ± 836 μM CH_4_) and TMA (9524 ± 3626 μM CH_4_, with a wide range of 5361–11 993 μM) on average more than the MMA controls, consistent with what has been observed for other methylotrophic methanogens [54]. Incubations amended with 10 mM methanol (MeOH) did not produce methane after 47 days of incubation at 70°C. Due to its use by sulfate-reducing organisms as an electron donor [55], 10 mM lactate (LAC) was tested, as well as 10 mM MMA with 10 mM LAC, but none of these incubations produced methane. Production of methane has not been observed in any attempted transfers where hydrogen (99.9999% purity) was present in the headspace, or hydrogen with MMA was added.

The enrichment grew optimally at both 64 and 70°C with relative amounts of methane produced at 5304 ± 451 μM and 5202 ± 606 μM, respectively. This deviates from the predicted optimal growth temperature of 74.4°C, which was derived from the translation of proteins in the *Ca*. M. hypatiae MAG using Tome [56]. This is lower than the observed range of growth and optimum temperatures for type strains of non-methanogenic *Archaeoglobus* which have been demonstrated to grow between 50 and 95°C with optimal temperatures between 75 and 83°C in organisms sourced predominantly from deep sea vent environments [30]. No methane production was detected at temperatures 77°C or above or lower than 64°C after 47 days of incubation (Dataset S4).

### Genomic and transcriptomic basis for methanogenesis

**Figure 5:**
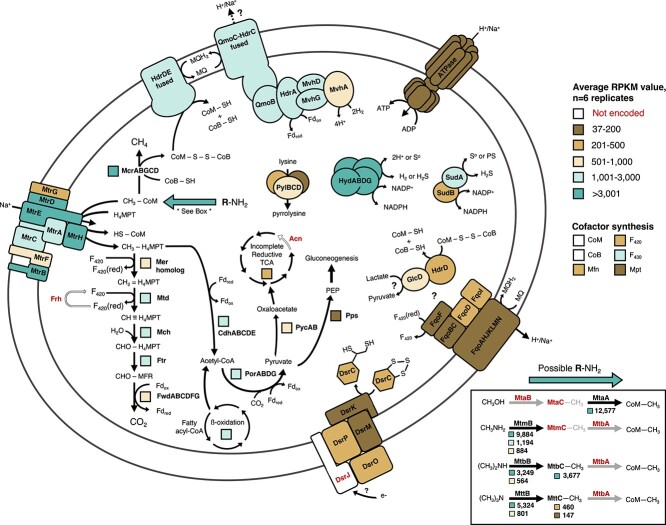
Transcriptional activity in *Ca*. M. hypatiae grown under methanogenic conditions (N_2_ headspace, 10 mM MMA, and 0.01% yeast extract); transcriptionally active proteins are shown in bold black font; proteins not encoded in the MAG are colored in white and denoted in bold red font; average RPKMs values of six biological replicates are depicted; RPKM values are represented by boxes or colored subunits close to each protein and are colored according to their expression level with the RPKM value of the lowest expressed gene depicted, 37 RPKM; for enzymes comprising multiple subunits, the beta-oxidation pathway, and the TCA cycle, an average RPKM value representing the transcribed enzymes is used. *Ca*. M. hypatiae is transcriptionally active under methanogenic conditions and encodes the ability to convert methyl-groups from mono-, di-, and TMA to methane; this ability is enabled by several copies of substrate-specific methyltransferases and corrinoid proteins highlighted in the box to the bottom right; a complete list of genes described in this figure, their transcription levels, and their abbreviations is provided in Dataset S5.

The assembled metagenome obtained at the end of the SIT experiment was used to align a total of 23 376 154 metatranscriptome mRNA reads obtained from six replicates harvested in the exponential growth phase and to create a detailed reconstruction of the metabolism of *Ca*. M. hypatiae (Figs 3A and B and 5, Dataset S5). A total of 22 891 651 reads, i.e. 97.8% of all recovered reads, were recruited to the *Ca*. M. hypatiae MAG. Only 2.1% of the total mRNA reads (484503) were aligned with other co-enriched organisms. Among these, only 13 genes across four MAGs were expressed above 200 RPKMs and just five genes exceeded >1000 RPKM. Genes required for the conversion of methylamine to methane were among the top 2% of highest expressed genes transcribed by *Ca*. M. hypatiae, including genes encoding the MCR complex (*mcrAGCDB*; 13 046–18 098 RPKM), one of three MMA methyltransferase copies (*mtmB*; 9884 RPKM), DMA corrinoid (*mtbC*; 3677 RPKM), and methanol:coenzyme M methyltransferase (*mtaA*; 12 577 RPKM) (Fig. 5). Seven copies of substrate-specific methyltransferases for MMA (*mtmB*; 3 copies), DMA (*mtbB*; 2 copies), and TMA (*mttB*; 2 copies) were present in the genome, but methanol methyltransferase (*mtaB*) was not identified. These genes were differentially expressed with one copy for each type of methylamine expressed above 3200 RPKM. In addition to *mtbC*, two gene copies of the TMA corrinoid protein (*mttC*) were found in the genome but their expression was relatively low (<460 RPKM average). MMA corrinoid (*mtmC*) or methanol corrinoid (*mtaC*) proteins were not identified in *Ca*. M. hypatiae. Additionally, genes were expressed for pyrrolysine synthesis (*pylBCD*; 819, 343, 37 RPKM) and the methyltransferase corrinoid activation protein (*ramA*; 1076 RPKM), both of which support methylamine methyltransferases in methylotrophic methanogenesis [57, 58]. The absence of *mtmC* and the high expression levels of *mtbC* (3677 RPKM) and *mtaA* (12 577 RPKM) suggest that they are responsible for the transfer of a methyl group from MMA to coenzyme M (CoM) after it has been transferred by a substrate-specific methyltransferase (*mtmB*). Consistent with the observed methane production from DMA and TMA, *Ca*. M. hypatiae can use these methylamines and expressed the corresponding genes (*mtbB*, *mttB*) at comparatively high levels (JOOIALLP_01813 *mtbB* 3249 RPKM; JOOIALLP_01787 *mttB* 5324 RPKM; Figs 4B and 5). It is worth noting that the expression of mtbB/mttB was detected despite the culture not having been previously exposed to DMA or TMA at the time of the transcriptomics experiment. We hypothesize that *Ca*. M. hypatiae could employ one of two strategies: it either (i) constitutively expresses all substrate-specific methyltransferases and corrinoid proteins as a precautionary measure to accommodate substrates potentially encountered *in situ*, or (ii) *Ca*. M. hypatiae transcriptionally co-regulates the genes responsible for these functions.

*Ca*. M. hypatiae expressed the methyl-branch of the WLP and the acetyl-CoA decarbonylase/synthase complex (Cdh, *cdhABCDE*), which is consistent with genes observed and shown to be expressed in sulfate-reducing *Archaeoglobi* genomes [55]. This includes two paralogous copies of 5,10-methylenetetrahydromethanopterin reductase (*mer*) which might function as a traditional Mer, considering that these genes are also members of the large luciferase-like monooxygenase family (pfam00296) [35]. The expression of genes in the WLP varied. Methylenetetrahydromethanopterin dehydrogenase (*mtd*), methenyltetrahydromethanopterin cyclohydrolase (*mch*), formylmethanofuran-tetrahydromethanopterin N-formyltransferase (*ftr*), formylmethanofuran dehydrogenase (*fwdABC*), and one copy of the *mer* homologs were expressed at comparatively high levels (456–2763 RPKM), whereas FwdDEFG and the other *mer* copy were only minimally expressed (<180 RPKM) (Dataset S5). The high expression of the Cdh complex (*cdhACDE*; 3063 ± 362, *cdhB* 677 RPKM average across subunits) suggests that *Ca*. M. hypatiae is capable of autotrophically fixing CO_2_ to acetyl-CoA as has been shown for other *Archaeoglobus* species [59]. Acetyl-CoA could also be derived from the degradation of fatty acids present in yeast extract through the process of beta-oxidation. Enzymes involved in this pathway were expressed at moderate to high levels during growth (Dataset S6). Pyruvate synthase (Por) was highly expressed providing a way for acetyl-CoA to be converted to pyruvate and subsequently be fed into major biosynthetic pathways. Specifically, *Ca*. M. hypatiae encodes pyruvate carboxylase (PycAB), an incomplete reductive tricarboxylic acid cycle (rTCA), phosphoenolpyruvate synthase (Pps), most enzymes needed for gluconeogenesis, and several enzymes associated with the pentose phosphate pathway in archaea, which were all expressed at varying levels (Dataset S5). Together, these pathways provide *Ca*. M. hypatiae the capacity to synthesize amino acids, carbohydrates, integral components of the cell wall, and vital sugars for nucleic acids.

Several complexes related to energy conservation and electron transport were moderately to highly expressed. *Ca*. M. hypatiae encodes a fused heterodisulfide reductase (*hdrDE*) that was highly expressed (1106 ± 120 RPKM) in addition to a fused *hdrD/mvhD* and four copies of *hdrD* that were all expressed at much lower levels (<500 RPKM). The differing levels of transcription suggest that the membrane-bound HdrDE is responsible for the regeneration of coenzymes M and B through the reduction of heterodisulfide (CoM-S-S-CoB). Additionally, the absence of HdrB, which contains the active site for disulfide reduction, eliminates the possibility that disulfide reduction could occur via a HdrABC complex [60]. As reported for *Ca*. M. nevadensis [35], a unique gene cluster was identified containing F_420_-non-reducing hydrogenase (MvhAGD), two HdrA copies and a QmoC fused to a HdrC. One HdrA copy (JOOIALLP_01710) was predicted by DiSCo analysis as a quinone-modifying oxidoreductase (QmoB), a protein related to the HdrA of methanogens [61, 62]. This cluster was expressed at high levels (995–2431 RPKM average), suggesting its importance for electron transfer in *Ca*. M. hypatiae. We hypothesize that these subunits are associating together *in vivo* to bifurcate electrons from hydrogen (H_2_) to reduce both menaquinone (MQ) and ferredoxin (Fd_ox_), as proposed recently [35, 63]. Lastly, *Ca*. M. hypatiae moderately expressed a membrane-bound F_420_H_2_:quinone oxidoreductase (Fqo) complex (88–280 RPKM across subunits) and a V-type ATP synthase (24–442 RPKM across subunits).

The electrons required for reducing the CoM-S-S-CoB heterodisulfide could originate from two possible routes. The first possibility would rely on sourcing electrons from hydrogen, which could be oxidized by the Mvh-Qmo-Hdr complex coupled to MQ reduction. H_2_ may be produced through the activity of a group 3b [NiFe]-sulfhydrogenase (HydABDG), which was the highest expressed hydrogenase complex with an average RPKM of 4421 across subunits [64, 65]. To evolve hydrogen via HydABDG, reducing power, via NADPH, could be supplied by sulfide dehydrogenase (SudAB; SudA, 1088 RPKM; SudB, 495 RPKM). Alternatively, NADPH could instead be provided to biosynthesis pathways and therefore be decoupled from methanogenic metabolism. H_2_ could also potentially be sourced from fermentative bacteria in the enrichment culture; however, the low number of hydrogenases encoded by co-enriched organisms was only very lowly expressed at the time of sampling for metatranscriptomics (<51 RPKM). At this point, the source of H_2_*Ca*. M. hypatiae uses remains uncertain, as no H_2_ was added to the headspace. The second option for reducing the CoM-S-S-CoB heterodisulfide involves a hydrogen-independent electron transport system, where reduced F_420_ and ferredoxin are generated through the dismutation of methylated substrate to CO_2_ via the WLP. Reduced F_420_ could be oxidized by the Fqo complex and contribute to a reduced MQ pool that could be used by the fused HdrDE complex to reduce CoM-S-S-CoB. Reduced ferredoxin could be oxidized at a soluble FqoF to reduce F_420_ or at an Fqo complex lacking FqoF to reduce MQ [66, 67]. Based on the low expression levels of the Fqo complex (171 ± 67 RPKM) and the absence of F_420_-reducing hydrogenase (*frh*) from the genome, it is not likely the WLP runs in the reductive direction as a source of reduced F_420_ would be required. Resolving the exact configuration of the electron transport system encoded by *Ca*. M. hypatiae will require biochemical confirmation in future investigations.

Genes necessary for dissimilatory sulfate reduction typically observed in sulfate-reducing members of the *Archaeoglobi*, including dissimilatory sulfite reductase (*dsrAB*), sulfate adenylyltransferase (*sat*), and adenylylsulfate reductase (*aprAB*), were neither identified in the genome of *Ca*. M. hypatiae nor in the unbinned fraction of the metagenome. They were also absent from the comparatively incomplete MAG of *Ca*. M. nevadensis GBS [35]. However, *Ca*. M. hypatiae encodes subunits *dsrMK* and *dsrOP* of the Dsr complex in addition to *dsrC*. This complex is strictly conserved in sulfate-reducing organisms [68] where it mediates electron transfer from the periplasm to the cytoplasm reducing the disulfide bond found in DsrC cysteines [69]. The expression of the Dsr complex and *dsrC* was low (450 ± 63 RPKM) during growth on MMA suggesting it is not vital to the metabolism of *Ca*. M. hypatiae. The presence of the Dsr complex, DsrC, and subunits QmoC and QmoB in the genome may be explained as evolutionary remnants from ancestral *Archaeoglobi*, growing initially as sulfate-reducing organisms but later transitioning to a methanogenic lifestyle [7, 8]. This raises the question whether intermediate of this process, *Archaeoglobi* capable of both methanogenesis and sulfate-reduction (and possible anaerobic oxidation of methane), still exist today [25, 28].

Collectively, the metagenomic and transcriptomic data confirmed that *Ca*. M. hypatiae is not only the sole archaeon but the sole methanogen in our culture. The metabolic reconstruction and metatranscriptomic results are consistent with methylotrophic methanogenesis from methylamines. The absence of genes required for sulfate reduction eliminates the possibility for this metabolism in *Ca*. M. hypatiae. A unique gene cluster (Mvh-Qmo-Hdr) potentially involved in energy conservation was expressed; however, future studies will be required to test how *Ca*. M. hypatiae internally cycles electrons for methanogenesis and if it sources H_2_, or other reductants, from the medium or co-enriched bacteria.

### Distribution of *Ca*. methanoglobus across geothermal features in YNP

16S rRNA and *mcrA* gene amplicon sequence data generated in a recent microbial diversity survey of 100 geothermal features in YNP [33] were used to analyze the distribution of *Archaeoglobi* related to *Ca*. M. hypatiae (SI Appendix, Fig. S5). 16S rRNA gene amplicons closely related to *Ca*. M. hypatiae (96.7%–100% sequence identity) were found in seven DNA samples from six hot springs (pH 5.1–9.35, 31–78°C) in addition to hot spring LCB024 (the source of this culture) at relative abundances ranging from 0.02% to 0.22%. In addition, *mcrA* gene ASVs affiliated with Archaeoglobi were PCR-amplified from 53 DNA samples, out of 201 total samples that had been screened by PCR. These 53 samples had been collected from microbial mats or sediments originating from 36 geothermal features distributed across various thermal regions within YNP by Lynes *et al*. [33]. Archaeoglobi-related *mcrA* genes were found in geothermal features with a pH range of 2.61 to 9.32 and a temperature range of 18.4–93.8°C. Collectively, our results and the studies by Wang *et al*. and Buessecker *et al*., who reported that Mcr-encoding *Archaeoglobi* are present [35] and transcriptionally active in hot spring mesocosms [34], demonstrate the previously overlooked role that *Archaeoglobi* might play in the anaerobic carbon cycle of geothermal environments.

## Conclusion

The cultivation of *Ca*. M. hypatiae LCB24 provides direct experimental evidence that members of the *Archaeoglobi* are methanogens. *Ca*. M. hypatiae can use MMA, DMA, and TMA as methanogenic substrates and grows optimally at 64–70°C, as evidenced by metagenomics, metatranscriptomics, and isotope tracing experiments. Metagenomic sequencing and phylogenomic analysis confirmed the close relationship of *Ca*. M. hypatiae to other Mcr-encoding *Archaeoglobi* and the relatedness of its *mcrA* to MAGs of the TACK superphylum, some of which have recently been shown to also be methanogens [70, 71]. Together, this supports the idea that the capacity for methanogenesis is deeply rooted in the archaea and possibly dates to the last common ancestor of archaea [1, 3, 7, 8, 72]. The wide distribution of *Archaeoglobi*-affiliated *mcrA* gene sequences and *Ca*. M. hypatiae-related 16S rRNA gene sequences in geothermal features across YNP suggests that members of this lineage play a hitherto unaccounted-for role in anaerobic carbon cycling in these extreme ecosystems. Future studies of *Ca*. M. hypatiae and other methanogens will provide valuable insights into the evolution of methane metabolism and the significance of these archaea in biogeochemical cycles across geothermal and other environments.

## Supplementary Material

supplementary_materials_wrae026

## Data Availability

All metagenomic, metatranscriptomic, and amplicon data discussed in this manuscript are available under NCBI BioProject ID PRJNA1014417. McrA gene amplicon data from YNP hot springs discussed in this manuscript have been previously published (Lynes *et al*.) and are available under NCBI under BioProject PRJNA859922.
