# Supplementary material for: Methylotrophic methanogenesis in the Archaeoglobi revealed by cultivation of Ca. Methanoglobus hypatiae from a Yellowstone hot spring
Source: ISME J. 2024 Mar 7;18(1):wrae026. doi: 10.1093/ismejo/wrae026 (PMC10945360; doi:10.1093/ismejo/wrae026)
Supplement: supplementary_materials_wrae026 [file supplementary_materials_wrae026.zip › supplementary_materials_wrae026/SI text_track_OFF-final.pdf]

Supporting Information

SI Results and Discussion

Protologue

Methanoglobus hypatiae sp. nov.

Me.tha.no.glo.bus. Gr. pref. *methano-*, pertaining to methane; L. masc. n. -*globus*, sphere; Gr.L. masc. n. *Methanoglobus*, methane producing organism spherical in shape. This genus was named by Buessecker *et al.* (17). Hy.pa.ti.ae. Gr. fem. hypatiae, to honor Hypatia of Alexandria, a respected and renowned philosopher of ancient Alexandria, Egypt, who made significant contributions to the understanding of mathematics and astronomy. A symbol of intellectual courage and scholarly achievement. This archaeon was cultured from an unnamed hot spring in the Lower Culex Basin of Yellowstone National Park identified as feature LCB024 (1). This archaeon is an obligately anaerobic thermophile that performs methylotrophic methanogenesis using methylamines and grows as regular to irregular coccoid cells approximately 0.5 to 1 µm in width. The type genome of this archaeon is deposited at NCBI under BioProject PRJNA1014417, accession number will be added upon publication.

SI Materials and Methods

Amplicon Sequencing and Analysis

DNA was extracted from environmental slurry samples and enrichment cultures sampled on the day of transfer using the FastDNA Spin Kit for Soil (MP Biomedicals, Irvine, CA) following the manufacturer's guidelines. Archaeal and bacterial 16S rRNA genes were amplified with the updated Earth Microbiome Project primer set 515F and 806R (18). Amplicon libraries were prepared as previously described (1) and sequenced by the Molecular Research Core Facility at Idaho State University (Pocatello, ID) using an Illumina MiSeq platform with 2 x 250 bp paired end read chemistry. Gene reads were processed using QIIME 2 version 2022.8 (19). Primer sequences were removed from demultiplexed reads using cutadapt (20) with error rate 0.12 and reads truncated (130 bp forward, 150 bp reverse), filtered, denoised and merged in DADA2 with default settings (21). Processed 16S rRNA gene amplicon sequence variants (ASVs) were taxonomically classified with the sklearn method and the SILVA 138 database (22). The R package *decontam* (version 1.18.0) (23) was used to remove contaminants using the "Prevalence" model with a threshold of 0.5.

Annotation and Reconstruction of Metabolic Potential

Genes associated with methanogenesis pathways, dissimilatory sulfur metabolism pathways, coenzyme and cofactor biosynthesis, energy conservation, and beta-oxidation, were inventoried. Annotations assigned by Prokka were refined through manual evaluation using KofamKOALA, NCBI BLASTP, NCBI's Conserved Domain Database, InterPro, the hydrogenase classifier HydDB, and DiSCo (24-29).

Phylogenetic and Phylogenomic Analyses

Average nucleotide identities (ANI) of 16S rRNA genes were calculated with blastn, with ANI and average amino acid identities (AAI) calculated by pyani v02.2.12 (ANIb) and CompareM v0.0.23 (--fragLen 2000) (https://github.com/dparks1134/CompareM), respectively for selected Archaeoglobales genomes and MAGs (Table 1). Phylogenetic analysis of 16S rRNA genes was performed with fasttree (30) using masked alignments generated by ssu-align.

Archaeoglobales MAGs and reference genomes were screened for 54 phylogenetically informative single copy proteins (31, 32) of which a subset of 33 proteins were identified in them all (SI Appendix, Table S4). In order to maximize the number of proteins compared across references, MAGs LMO1 and LMO3 were excluded from this analysis, as they lacked 2 and 7 proteins out of the total 33, respectively. These were then aligned with muscle (33), concatenated, and phylogenomically analyzed with maximum likelihood analysis with fasttree (WAG model). McrA alignments were performed with MAFFT-LINSi v7.522 (34), trimmed with trimAL v1.4.rev22 (35) using a 0.5 gap threshold, and maximum likelihood trees were built with IQTree2 v2.0.6 (36) using LG+C60+F+G model and 1,000 ultrafast bootstraps.

Temperature and Substrate Optimum Experiments

Methane production and growth of Archaeoglobus was evaluated at different temperatures and in the presence of methylated substrates (i.e., methanol and mono-, di-, and trimethylamine), lactate, and media prepared without yeast extract. The sixth transfer of the enrichment was used to inoculate triplicate 30 mL serum bottles containing 15 mL of medium with 8% v/v inoculum, streptomycin (50 mg/L), vancomycin (50 mg/L), and 10 mM of each substrate tested. Cultures were evaluated at 60˚C, 64˚C, 70˚C, 77˚C, 80˚C, and 85˚C with 10 mM MMA. Separately, we tested whether the culture would grow on the following substrate (combinations): 10 mM dimethylamine (DMA); 10 mM trimethylamine (TMA); 10 mM methanol (MeOH); 10 mM lactate (LAC); 10 mM MMA and 10 mM LAC); 10 mM MMA with media without yeast extract; and a control in media without yeast or any methanogenic substrate. The 70˚C cultures amended with 10 mM MMA served as the control. All incubations were performed in biological triplicate.

Methane Measurements

During cultivation, 250 µL subsamples of the headspace were taken using a gas tight syringe (Hamilton) and injected into a 10 mL autosampler vial that had been sealed with grey chlorobutyl septa. Samples were taken from the autosampler vials and injected into a Shimadzu 2020-GC gas chromatograph equipped with a GS-CarbonPLOT column (30 m x 0.32 mm; 1.5 µm film thickness; Agilent) and a Rt-Q-BOND column (30 m x 0.32 mm; 1.5 µm film thickness; Restek) using helium as a carrier gas. All injections were performed by a Shimadzu AOC-6000 autosampler robot. The injector, column, and flame ionization detector (FID) were maintained at 200˚C, 50˚C, and 240˚C, respectively. Methane concentrations were calculated based on injection of a standard curve.

Fluorescence *in situ* hybridization and cell counts

Aliquots of enrichment cultures incubated with ^{13}C-MMA during the SIT experiment were treated with 2% paraformaldehyde (PFA) and fixed for 1 hr at room temperature. Following fixation, cells were washed twice with 1x PBS, followed by centrifugation at 16,000 × g to remove the supernatant, resuspended in 1x PBS, and stored at 4˚C. For direct cell counts, aliquots of fixed cell suspensions were filtered onto polycarbonate filters (0.2 µm pore size, 25 mm diameter, GTTP Millipore, Germany) and air dried before filter pieces were cut and embedded in 0.2% low melting agarose. We attempted to use the Archaeoglobales-specific probe Arglo32 (37), however fluorescent signal was insufficient. Given *Ca.* M. hypatiae was the sole archaeon in the enrichment culture, the relative abundance of *Ca.* M. hypatiae cells was determined via catalyzed reporter deposition fluorescence *in situ* hybridization (CARD-FISH) using the general archaea-targeted 16S rRNA oligonucleotide probe Arch915 (38). Total cell counts were based on DNA-stained cells using DAPI (4,6-diamidino-2-phenylindole). CARD-FISH was performed as previously described (39). Cell wall permeabilization was achieved with a brief treatment of 0.1 M HCl (1

min, RT) followed by treatment with 0.01 M HCl (15 min, RT). Endogenous peroxidases were inactivated with 0.15% H_2O_2 in methanol (30 min, RT). A formamide concentration of 35% was used for all hybridization reactions (2.5 hrs, 46˚C). CARD was performed using Alexa Fluor 594 labeled tyramides for 30 min at 46˚C. Following signal amplification, an additional washing step in 1x PBS was included to reduce background fluorescence (15 min, RT, dark). Samples were stained with DAPI, embedded in Citifluor-Vectashield, and enumerated using an epifluorescence microscope (Leica DM4B).

Scanning electron microscopy (SEM)

An aliquot of the enrichment culture at transfer 7 (T7) was treated with 2% paraformaldehyde (PFA) and fixed for 1 hr at room temperature. Following fixation, cells were washed twice through centrifugation at 16,000 × g to remove the supernatant, resuspended in 1x phosphate buffered saline (PBS), and stored at 4˚C. Samples for imaging were prepared according to Schaible et al., 2022 (40). Briefly, a square coupon of mirror-finished 304 stainless steel (25 mm diameter, 0.6 mm thickness) was purchased from Stainless Supply (Monroe, NC). The coupon was cleaned by washing with a 1% solution of Tergazyme (Alconox, New York, NY) and rinsed with Milli-Q water. The coupon was dried under compressed air and stored at room temperature. 5 µL of fixed sample was spotted on the coupon and air-dried at 46 °C for 3 min. The coupon was then dried for 1 m each step in a successive ethanol series starting with 10% ethanol and increasing by increments of 10% with the last step 90% ethanol. SEM images were captured using a Zeiss (Jena, Germany) SUPRA 55VP field emission scanning electron microscope (FE-SEM). The microscope was operated at 1 keV under a vacuum of 0.2–0.3 mPa, with a working distance of 5.4-6.2 mm at the Imagining and Chemical Analysis Laboratory (ICAL) of Montana State University (Bozeman, MT). No conductivity coating was applied before SEM analysis as the microscope was operated at 1 keV.

SI Tables

Table S1. Extended community composition history of methanogenic enrichment cultures via estimated relative abundance (%) from 16S rRNA gene amplicon sequencing.

	**LCB024 sediment**	**Slurry 11/2021**	**Slurry 02/2022**	**Initial Enrichment**	**T1**	**T2**	**T3**	**T4**	**T4 - MG**	**T5**	**SIT-MG**
Archaeoglobaceae ASV_78ad2	0.46	0.32	0.02	6.42	74.8	68.9	48.5	46.0	84.7	62.2	92.8
Pseudothermotoga ASV_17231	2.14	1.06	1.08	18.93	6.8	17.1	28.5	37.4	14.5	15.7	3.2
Other Archaeoglobaceae uncultured	0.00	0.00	0.00	1.54	0.0	0.01	0.0	0.0	0.0	0.0	0.0
Confirmed methanogenic archaea	0.25	0.38	0.45	0.00	0.0	0.0	0.0	0.0	0.0	0.0	0.0
Other Archaea	13.51	11.87	10.49	2.67	0.0	0.0	0.0	0.04	0.0	0.0	0.0
Other Bacteria	83.63	86.37	87.95	70.44	18.4	14.1	23.1	16.5	0.8	22.1	4.0

Table S2. Methane production of the enrichment culture over time. T, transfer.

Culture ID	Days continuous	Days	CH_4 (µM)	CH_4 (mM)	CH_4 (ppm)	CH_4 (%)
**1A – initial enrichment**	6	6	4.91	0.00	118.67	0.01
	13	13	16.73	0.02	404.73	0.04
	20	20	12.98	0.01	314.13	0.03
	27	27	12.86	0.01	311.20	0.03
	36	36	32.92	0.03	796.48	0.08
	48	48	146.28	0.15	3538.80	0.35
	53	53	279.15	0.28	6753.17	0.68
	63	63	419.11	0.42	10139.33	1.01
	70	70	447.12	0.45	10816.36	1.08
**T1**	82	12	14.40	0.01	348.40	0.03
	91	21	50.85	0.05	1230.06	0.12
	98	27	198.41	0.20	4800.09	0.48
	106	35	390.47	0.39	9445.97	0.94
	113	42	565.32	0.57	13676.04	1.37
	117	46	688.44	0.69	16654.06	1.67
	126	55	1442.03	1.44	34885.74	3.49
	133	62	1844.13	1.84	44611.75	4.46
**T2**	146	7	15.22	0.02	368.32	0.04
	158	18	16.11	0.02	389.83	0.04
	175	34	49.40	0.05	1195.06	0.12
	197	57	680.54	0.68	16463.41	1.65
	221	81	2200.24	2.20	53227.90	5.32
	229	89	2654.34	2.65	64213.66	6.42
	238	98	2230.72	2.23	53964.66	5.40
**T3**	243	5	18.17	0.02	439.53	0.04
	259	21	10.70	0.01	258.82	0.03
	266	28	536.13	0.54	12969.71	1.30
	274	36	2413.23	2.41	58379.47	5.84
	280	42	2283.95	2.28	55253.34	5.53
	284	46	2193.96	2.19	53077.04	5.31
**T4**	294	10	17.36	0.02	420.07	0.04
	301	17	18.12	0.02	438.37	0.04
	309	25	12.51	0.01	302.54	0.03
	319	35	1944.01	1.94	47028.08	4.70
	327	43	3054.00	3.05	73881.94	7.39
	335	51	2619.45	2.62	63370.12	6.34
**T5**	347	10	13.41	0.01	324.42	0.03
	355	18	13.14	0.01	317.78	0.03
	365	28	137.59	0.14	3328.60	0.33
	371	34	1023.35	1.02	24756.27	2.48
	375	38	2218.75	2.22	53674.81	5.37
	384	46	2459.29	2.46	59493.85	5.95
**T6**	386	2	16.29	0.02	394.19	0.04
	393	9	21.33	0.02	516.14	0.05
	400	16	18.68	0.02	451.92	0.05
	407	23	245.86	0.25	5947.76	0.59
	412	28	2555.27	2.56	61815.61	6.18
	414	30	3432.93	3.43	83051.24	8.31
	416	32	3621.90	3.62	87623.79	8.76
	418	34	3942.97	3.94	95385.88	9.54
**T7**	428	9	34.33	0.03	830.38	0.08
	435	16	296.91	0.30	7182.55	0.72
	440	21	2931.05	2.93	70907.06	7.09
	444	25	3164.45	3.16	76553.52	7.66
	446	27	3338.04	3.34	80751.55	8.08

Table S3. Extended Archaeoglobales metagenome assembled genome and isolate genome statistics (1-16). A combined assembly of metagenomes from T4-MG and SIT-MG (Fig. 1A) for the *Ca.* M. hypatiae LCB24 MAG was used as it yielded an improved assembly. GTDB classified the YNP, GBS, and China MAGs as: d_Archaea;p_Halobacteriota;c_Archaeoglobi;o_Archaeoglobales; f_Archaeoglobaceae;g_WYZ-LMO2;s_WYZ-LMO2. Len., length; Compl., completeness; Redun, redundancy; Strain Hetero., strain heterogeneity; pOGT, predicted optimal growth temperature. [*] stop codon interrupts *mcrA* sequence; [a] Both sequences 5′ start; not identical; [b] Consists of 1 chromosome and 1 plasmid.

	Seqs	Len (Mb)	GC (%)	Compl. (%)	Redun. (%)	16S	tRNA	CDS	*mcrA*	CRISPRs	pOGT (°C)	Citation
***Ca.* M. hypatiae LCB24**	19	1.624	46.12	100	1.31	1	44	1,760	1	2	74.44	This study
Archaeoglobales LCB024-003	179	1.157	45.72	88.48	1.31	0	31	1,226	1	1	73.90	Lynes *et al.* 2023
Archaeoglobales WYZ-LMO2	220	1.514	45.92	97.60	0	0	40	1,614	1	2	72.40	Wang *et al.* 2019
Ca. M. hydrogenotrophicum bin74	127	1.547	45.63	92.81	1.31	1	34	1,679	0[*]	9	75.32	Liu *et al.* 2020
Archaeoglobales SJ3.Bin34	73	1.468	46.00	98.69	0.65	0	39	1,581	1	2	73.13	Colman *et al.* 2019
Ca. Methanoglobus nevadensis GBS	71	1.602	47.16	98.04	3.66	1	47	1,785	1	2	74.75	Peacock *et al.* 2013
Archaeoglobales GMQP bin32	20	1.727	41.26	100	0.98	1	39	1,931	1	2	71.88	Hua *et al.* 2019
Archaeoglobales GMQP_D bin 18	22	1.565	42.01	97.39	0.98	1	45	1,747	1	3	69.41	Wang *et al.* 2023
Archaeoglobales JZ-3 D bin 138	32	1.550	42.08	99.35	1.31	1	43	1,701	1	2	69.11	Wang *et al.* 2023
Archaeoglobales WYZ-LMO1	140	1.557	43.85	88.89	1.31	1	41	1,701	1	1	72.72	Wang *et al.* 2019
Archaeoglobales WYZ-LMO3	135	1.568	43.93	88.03	1.96	2[a]	35	1,670	1	2	73.09	Wang *et al.* 2019
Ca. M. hydrogenotrophicum bin11	252	1.220	47.59	91.83	6.17	1	27	1,387	1	0	72.64	Liu *et al.* 2020
Ca. M. hydrogenotrophicum bin16	46	1.668	45.49	96.51	1.31	0	41	1,864	1	2	72.69	Liu *et al.* 2020
Archaeoglobus JZ bin24	35	1.492	44.66	99.35	1.31	0	44	1,642	0	2	68.52	unpublished
Archaeoglobales LCB024-002	66	1.334	42.28	97.39	0.03	0	41	1,459	0	0	73.46	Lynes *et al.* 2023
Archaeoglobales LCB003-04	128	1.335	42.27	96.73	1.96	0	38	1,447	0	0	73.71	Lynes *et al.* 2023
Archaeoglobales SJ3.bin61	45	1.270	41.68	89.54	0	1	40	1,392	0	0	73.34	Colman *et al.* 2019
Archaeoglobales JZ_75 SW bin109	16	1.562	39.48	99.35	0.65	1	45	1,762	1	5	74.34	Wang *et al.* 2023
Archaeoglobus fulgidus DSM 4304	1	2.178	48.58	100	0	1	46	2,440	0	3	79.88	Klenk *et al.* 1997
Archaeoglobus neptunius SE56	32	2.116	46.05	100	0	1	47	2,336	0	2	78.26	Slobodkina *et al.* 2021
Archaeoglobus profundus DSM 5631	2[b]	1.563	46.05	100	0	1	48	1,784	0	0	85.86	von Jan *et al.* 2010
Ferroglobus placidus DSM 10642	1	2.196	44.14	100	0	1	49	2,467	0	1	83.83	Anderson *et al.* 2011
Geoglobus acetivorans SBH6	1	1.861	46.84	99.84	0	1	48	2,168	0	6	78.22	Mardanov *et al.* 2015
Geoglobus ahangari 234	1	1.770	53.11	100	0	1	46	1,985	0	7	84.11	Manzella *et al.* 2015
Archaeoglobus sulfaticallidus PM70-1	1	2.077	43.24	100	0	1	51	2,237	0	1	76.12	Stokke *et al.* 2013
Archaeoglobus veneficus SNP6	1	1.902	47.05	99.35	0	1	46	2,055	0	2	76.18	Mukherjee *et al.* 2017
Ca. Polytropus marinifundus rG16	21	2.129	40.22	99.84	1.96	1	44	2,286	2	0	66.39	Boyd *et al.* 2019

Table S4. Conserved single copy proteins used in phylogenomic analysis of MAGs and isolates.

arCOG	Gene	Product
arCOG00405	GRS1	Glycyl-tRNA synthetase (class II)
arCOG00779	RplO	Ribosomal protein L15
arCOG00785	RpmC	Ribosomal protein L29
arCOG01001	Map	Methionine aminopeptidase
arCOG01183	Kae1p/TsaD	Subunit of KEOPS complex, contains a domain with ASKHA fold and RIO-type kinase (AP-endonuclease activity)
arCOG01228	Ffh	Signal recognition particle GTPase
arCOG01722	RpsM/rps13p	Ribosomal protein S13
arCOG01758	RpsJ/rps10p	Ribosomal protein S10
arCOG04070	RplC	Ribosomal protein L3
arCOG04071	RplD	Ribosomal protein L4
arCOG04072	RplW	Ribosomal protein L23
arCOG04086	RpmD	Ribosomal protein L30
arCOG04087	RpsE	Ribosomal protein S5
arCOG04088	RplR	Ribosomal protein L18
arCOG04090	RplF/rpl6p	Ribosomal protein L6P
arCOG04091	RpsH/rps8p	Ribosomal protein S8
arCOG04094	RplX/rpl24p	Ribosomal protein L24
arCOG04095	RplN/rps14p	Ribosomal protein L14
arCOG04096	RpsQ/rps17p	Ribosomal protein S17
arCOG04097	RpsC/rps3p	Ribosomal protein S3
arCOG04098	RplV/rpl22p	Ribosomal protein L22
arCOG04113	RplP	Ribosomal protein L10AE/L16
arCOG04121	RnhB	Ribonuclease HII
arCOG04169	SecY	Preprotein translocase subunit SecY
arCOG04185	RpsO	Ribosomal protein S15P
arCOG04239	RpsD/rps4p	Ribosomal protein S4 or related protein
arCOG04242	RplM/rpl13p	Ribosomal protein L13
arCOG04243	RpsI/rps9p	Ribosomal protein S9
arCOG04245	RpsB/rps2p	Ribosomal protein S2
arCOG04255	RpsL/rps12p	Ribosomal protein S12
arCOG04256	RpoC/Rpo11	DNA-directed RNA polymerase subunit A''
arCOG04257	RpoC/Rpo3/rpoA1	DNA-directed RNA polymerase subunit A'
arCOG04277	Efp	Translation elongation factor P (EF-P)/translation initiation factor 5A (eIF-5A)

Table S5. Calculated cell density of replicates in the SIT experiment. FID measurements of replicates used to determine cell density. Density is calculated based on cell counts of DAPI and CARD-FISH labeled samples. Stdev., standard deviation. Letters A-F identify each replicate.

**Summary**	**$^{12}CH_4$**		**Cell Density, cells mL^{-1}**		
**Replicate ID**	**µM**	**ppm**	**DAPI**	**FISH**	**% labeled with Arch915**
5A Day 22	25	610	3.83×10^7	1.34×10^6	3.5
5B Day 22	57	1,374	1.88×10^7	5.07×10^6	12.7
5E Day 22	50	1,204	3.49×10^7	0	0
5F Day 22	132	3,196	4.59×10^7	2.21×10^6	4.8
**Day 22 Average**	66	1,596	3.45×10^7	2.16×10^6	5.3
**Day 22 Stdev.**	46	1,116	1.14×10^7	2.15×10^6	5.4
5A Day 32	741	17,917	4.26×10^7	2.85×10^7	66.9
5B Day 32	2,176	52,635	1.11×10^8	6.00×10^7	54.2
5E Day 32	1,779	43,035	3.40×10^7	1.74×10^7	51.2
5F Day 32	2,412	58,355	9.14×10^7	4.03×10^7	44.1
**Day 32 Average**	1,777	42,985	6.97×10^7	3.65×10^7	54.1
**Day 32 Stdev.**	739	17,868	3.73×10^7	1.82×10^7	9.6
5A Day 45	4,109	99,398	12.2×10^7	6.41×10^7	52.7

SI Figures

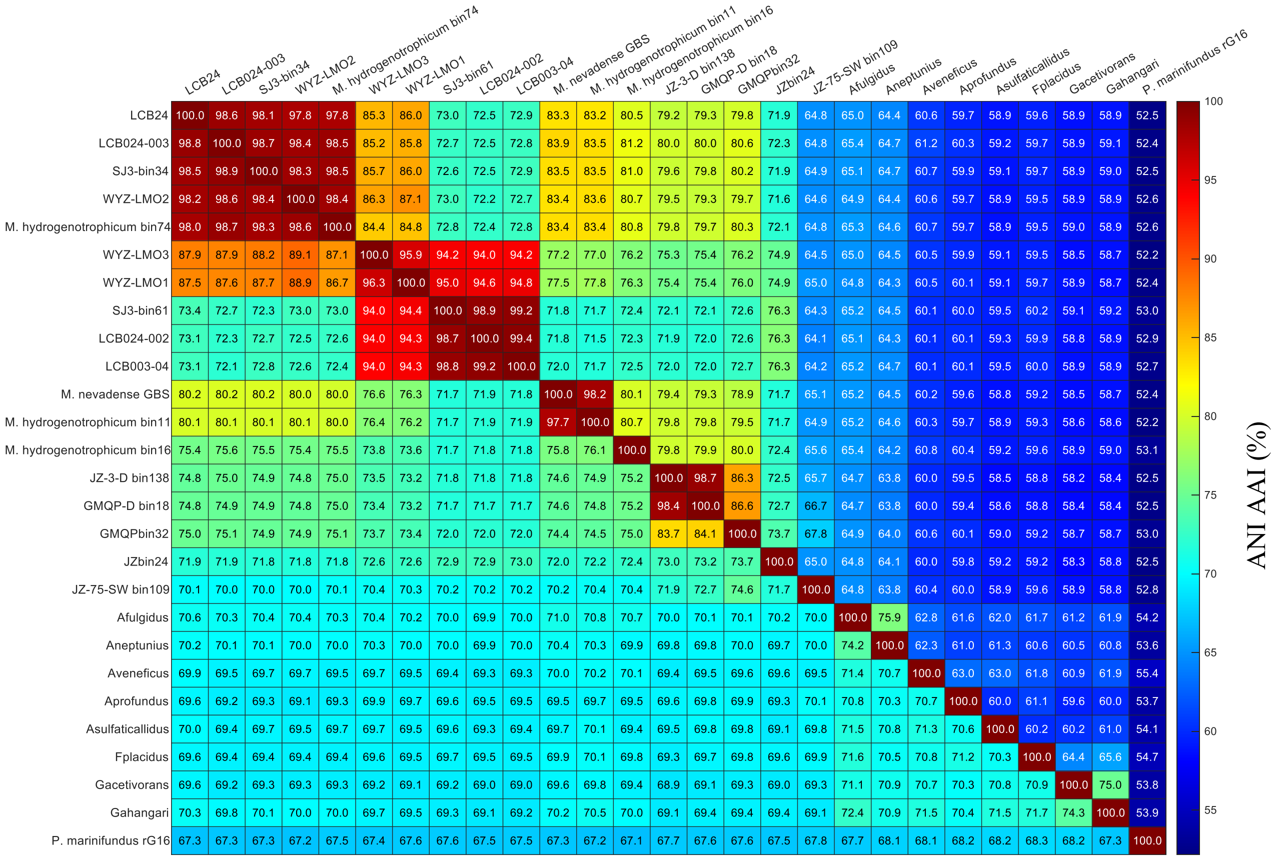

Fig. S1. Detailed ANI (lower half of matrix) and AAI (upper half of matrix) analysis of related Archaeoglobales MAGs and reference genomes.

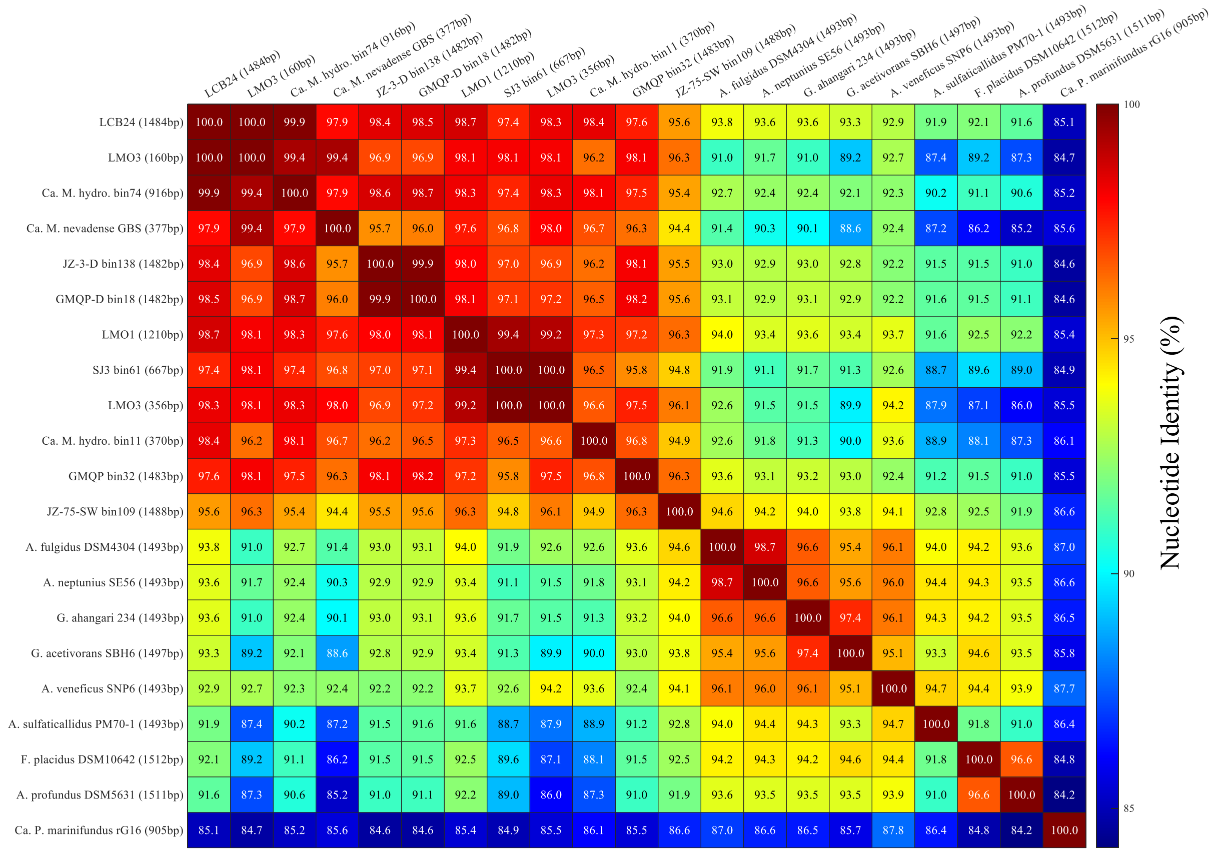

Fig. S2. 16S rRNA nucleotide identity analysis of closely related Archaeoglobales MAGs and reference genomes.

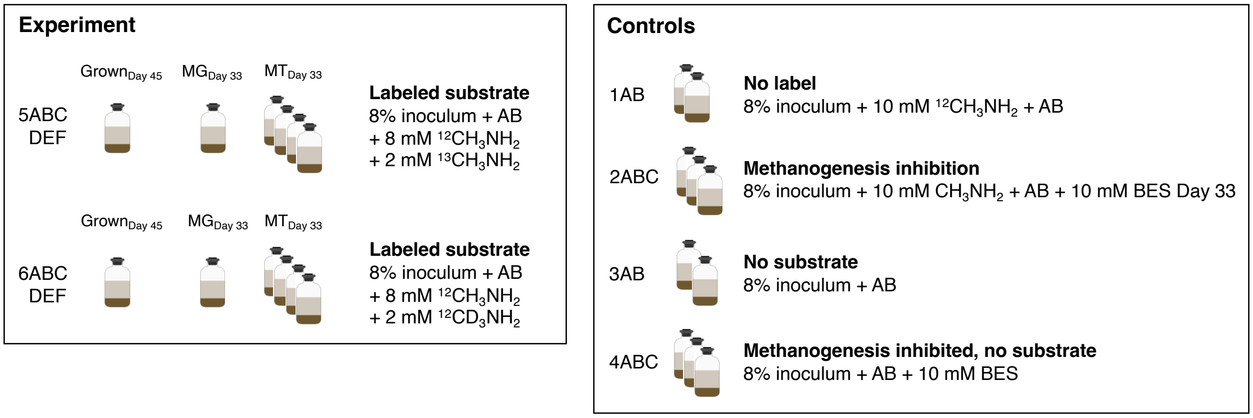

Fig. S3. Experimental setup of the stable isotope tracing (SIT) experiment. Incubations were carried out in 30 mL culture volumes in 60 mL serum bottles with 8% v/v inoculum, 50 mg/L streptomycin, 50 mg/L vancomycin, 10 mM MMA, and N_2 gas (99.999%) incubated in anoxic media (pH 7.8, 70˚C). Replicates sacrificed for analysis during mid-log phase are indicated. Of the eight samples harvested for metatranscriptomics, six were sequenced and used for analysis as two replicates did not yield sufficient RNA for sequencing. AB, antibiotics streptomycin and vancomycin, MG, metagenome sample; MT, metatranscriptome sample; BES, bromoethanesulfonate/methanogenesis inhibitor.

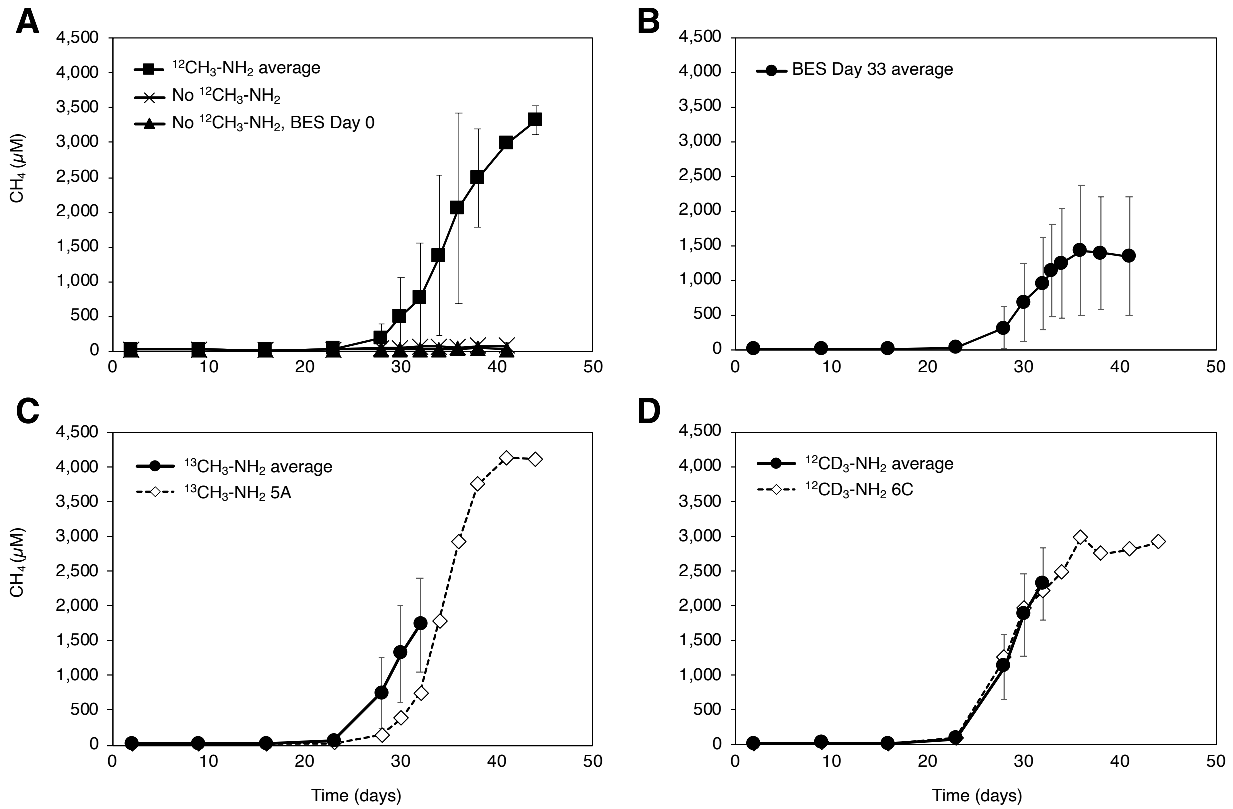

Fig. S4. $^{12}CH_4$ measurements by GC-FID during the stable isotope tracing experiment. Measurements can be found in Dataset S3.

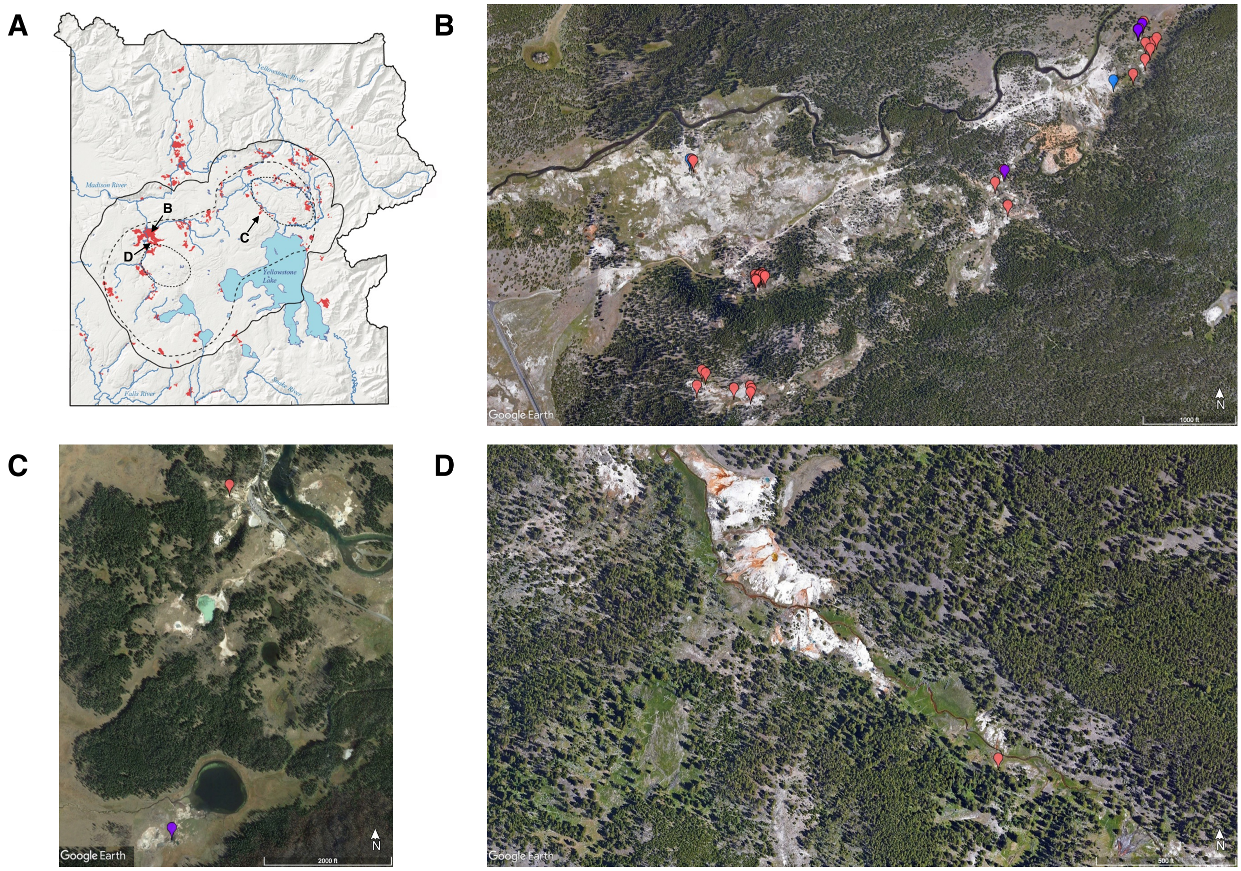

Fig. S5. Geographical distribution of geothermal features in Yellowstone National Park in which Archaeoglobi-related *mcrA* genes (n = 36) and *Ca.* M. hypatiae-related 16S rRNA genes (n = 6) were detected. Features are located in the (*A*) Map of Yellowstone National Park Wyoming, USA modified from Vaughan *et al.* 2014 (41) *(B)* Lower Culex Basin (n = 36), (*C*) Mud Volcano Region (n = 2), and (*D*) the White Creek Area (n = 1). These features spanned a wide pH (2.61-9.35) and temperature (18.4-93.8 ºC) range. Features in which *mcrA* were detected are marked in red and features with related 16S rRNA genes are shown in blue. Features in which both amplicons were detected are colored in purple. For details on these sites, their *mcrA* data, water geochemistry, and exact location, see Lynes & Krukenberg et al., 2023. Image source: Google Earth.

Description of Available Supplementary Datasets

SI Dataset S1. Extended metagenome assembled genome (SIT-MG) and isolate genome statistics. Seqs, sequences; avg_cov, average coverage; avg_gc, average G+C content; % rel. abund., percent relative abundance.

SI Dataset S2. GCMS measurements of masses 16 (CH_4), 17 ($^{13}CH_4$), and 19 ($^{12}CD_3H$) during the isotope tracing experiment. Percent of labeled methane is calculated as a fraction of provided labeled substrate. Stdev, standard deviation.

SI Dataset S3. Gas chromatograph FID measurements of $^{12}CH_4$ during isotope tracing experiment. NA, not available/measured.

SI Dataset S4. Gas chromatograph FID measurements of CH_4 during temperature optimum experiment. NA, not available/measured.

SI Dataset S5. Inventory of genes expressed by *Ca.* M. hypatiae LCB24 under methanogenic conditions and as depicted in Fig. 5. Expression levels averaged across six replicates are reported in reads per kilobase of transcript per million mapped reads (RPKM).

SI Dataset S6. Inventory of genes expressed by Ca. M. hypatiae LCB24 under methanogenic conditions belonging to the beta-oxidation pathway. Expression levels averaged across six replicates are reported in reads per kilobase of transcript per million mapped reads (RPKM).

References

1. Lynes MM, Krukenberg V, Jay ZJ, Kohtz AJ, Gobrogge CA, Spietz RL, et al. Diversity and function of methyl-coenzyme M reductase-encoding archaea in Yellowstone hot springs revealed by metagenomics and mesocosm experiments. ISME Commun. 2023;3:22.
2. Wang Y, Wegener G, Hou J, Wang F, Xiao X. Expanding anaerobic alkane metabolism in the domain of Archaea. Nat Microbiol. 2019;4:595-602.
3. Liu YF, Chen J, Zaramela LS, Wang LY, Mbadinga SM, Hou ZW, et al. Genomic and transcriptomic evidence supports methane metabolism in Archaeoglobi. mSystems. 2020;5:e00651-19.
4. Colman DR, Lindsay MR, Amenabar MJ, Boyd ES. The intersection of geology, geochemistry, and microbiology in continental hydrothermal systems. Astrobiology. 2019;19:1505-22.
5. Peacock JP, Cole JK, Murugapiran SK, Dodsworth JA, Fisher JC, Moser DP, et al. Pyrosequencing reveals high-temperature cellulolytic microbial consortia in Great Boiling Spring after in situ lignocellulose enrichment. PLoS One. 2013;8:e59927.
6. Hua ZS, Wang YL, Evans PN, Qu YN, Goh KM, Rao YZ, et al. Insights into the ecological roles and evolution of methyl-coenzyme M reductase-containing hot spring Archaea. Nat Commun. 2019;10:4574.
7. Wang J, Qu Y-N, Evans PN, Guo Q, Zhou F, Nie M, et al. Evidence for nontraditional *mcr*-containing archaea contributing to biological methanogenesis in geothermal springs. Science Advances. 2023;9:eadg6004.
8. Klenk H-P, Clayton RA, Tomb J-F, White O, Nelson KE, Ketchum KA, et al. The complete genome sequence of the hyperthermophilic, sulphate-reducing archaeon *Archaeoglobus fulgidus*. Nature. 1997;390:364-70.
9. Slobodkina G, Allioux M, Merkel A, Cambon-Bonavita MA, Alain K, Jebbar M, et al. Physiological and genomic characterization of a hyperthermophilic archaeon *Archaeoglobus neptunius* sp. nov. isolated from a deep-sea hydrothermal vent warrants the reclassification of the genus *Archaeoglobus*. Front Microbiol. 2021;12:679245.
10. von Jan M, Lapidus A, Glavina Del Rio T, Copeland A, Tice H, Cheng J-F, et al. Complete genome sequence of *Archaeoglobus profundus* type strain (AV18T). Stand Genomic Sci. 2010;2:327-46.
11. Anderson I, Risso C, Holmes D, Lucas S, Copeland A, Lapidus A, et al. Complete genome sequence of *Ferroglobus placidus* AEDII12DO. Stand Genomic Sci. 2011;5:50-60.
12. Mardanov AV, Slododkina GB, Slobodkin AI, Beletsky AV, Gavrilov SN, Kublanov IV, et al. The *Geoglobus acetivorans* genome: Fe (III) reduction, acetate utilization, autotrophic growth, and degradation of aromatic compounds in a hyperthermophilic archaeon. Appl Environ Microbiol. 2015;81:1003-12.
13. Manzella MP, Holmes DE, Rocheleau JM, Chung A, Reguera G, Kashefi K. The complete genome sequence and emendation of the hyperthermophilic, obligate iron-reducing archaeon "*Geoglobus ahangari*" strain 234T. Stand Genomic Sci. 2015;10:77.

14. Stokke R, Hocking WP, Steinsbu BO, Steen IH. Complete genome sequence of the thermophilic and facultatively chemolithoautotrophic sulfate reducer *Archaeoglobus sulfaticallidus* strain PM70-1T. Genome Announc. 2013;1:e00406-13.
15. Boyd JA, Jungbluth SP, Leu AO, Evans PN, Woodcroft BJ, Chadwick GL, et al. Divergent methyl-coenzyme M reductase genes in a deep-subseafloor Archaeoglobi. ISME J. 2019;13:1269-79.
16. Mukherjee S, Seshadri R, Varghese NJ, Eloe-Fadrosh EA, Meier-Kolthoff JP, Göker M, et al. 1,003 reference genomes of bacterial and archaeal isolates expand coverage of the tree of life. Nat Biotechnol. 2017;35:676-83.
17. Buessecker S, Chadwick GL, Quan ME, Hedlund BP, Dodsworth JA, Dekas AE. Mcr-dependent methanogenesis in Archaeoglobaceae enriched from a terrestrial hot spring. ISME J. 2023;17:1649-59.
18. Apprill A, McNally S, Parsons R, Weber L. Minor revision to V4 region SSU rRNA 806R gene primer greatly increases detection of SAR11 bacterioplankton. Aquat Microb Ecol. 2015;75:129-37.
19. Bolyen E, Rideout JR, Dillon MR, Bokulich NA, Abnet CC, Al-Ghalith GA, et al. Reproducible, interactive, scalable and extensible microbiome data science using QIIME 2. Nat Biotechnol. 2019;37:852-7.
20. Martin M. Cutadapt removes adapter sequences from high-throughput sequencing reads. EMBnetJ. 2011;17:10-2.
21. Callahan BJ, McMurdie PJ, Rosen MJ, Han AW, Johnson AJ, Holmes SP. DADA2: High-resolution sample inference from Illumina amplicon data. Nat Methods. 2016;13:581-3.
22. Quast C, Pruesse E, Yilmaz P, Gerken J, Schweer T, Yarza P, et al. The SILVA ribosomal RNA gene database project: improved data processing and web-based tools. Nucleic Acids Res. 2012;41:D590-D6.
23. Davis NM, Proctor DM, Holmes SP, Relman DA, Callahan BJ. Simple statistical identification and removal of contaminant sequences in marker-gene and metagenomics data. Microbiome. 2018;6:226.
24. Aramaki T, Blanc-Mathieu R, Endo H, Ohkubo K, Kanehisa M, Goto S, et al. KofamKOALA: KEGG Ortholog assignment based on profile HMM and adaptive score threshold. Bioinformatics. 2020;36:2251-2.
25. Lu S, Wang J, Chitsaz F, Derbyshire MK, Geer RC, Gonzales NR, et al. CDD/SPARCLE: the conserved domain database in 2020. Nucleic Acids Res. 2020;48:D265-D8.
26. Sondergaard D, Pedersen CN, Greening C. HydDB: A web tool for hydrogenase classification and analysis. Sci Rep. 2016;6:34212.
27. Blum M, Chang H-Y, Chuguransky S, Grego T, Kandasaamy S, Mitchell A, et al. The InterPro protein families and domains database: 20 years on. Nucleic Acids Res. 2021;49:D344-D54.
28. Neukirchen S, Sousa FL. DiSCo: a sequence-based type-specific predictor of Dsr-dependent dissimilatory sulphur metabolism in microbial data. Microb Genom. 2021;7(7).

29. Neukirchen S, Sousa FL. DiSCo: a sequence-based type-specific predictor of Dsr-dependent dissimilatory sulphur metabolism in microbial data. Microb Genom. 2021;7:000603.
30. Price MN, Dehal PS, Arkin AP. FastTree 2--approximately maximum-likelihood trees for large alignments. PLoS One. 2010;5:e9490.
31. Jay ZJ, Beam JP, Dlakic M, Rusch DB, Kozubal MA, Inskeep WP. Marsarchaeota are an aerobic archaeal lineage abundant in geothermal iron oxide microbial mats. Nat Microbiol. 2018;3:732-40.
32. Zaremba-Niedzwiedzka K, Caceres EF, Saw JH, Backstrom D, Juzokaite L, Vancaester E, et al. Asgard archaea illuminate the origin of eukaryotic cellular complexity. Nature. 2017;541:353-8.
33. Edgar RC. MUSCLE: multiple sequence alignment with high accuracy and high throughput. Nucleic Acids Res. 2004;32:1792-7.
34. Katoh K, Standley DM. MAFFT multiple sequence alignment software version 7: improvements in performance and usability. Mol Biol Evol. 2013;30:772-80.
35. Capella-Gutiérrez S, Silla-Martínez JM, Gabaldón T. trimAl: a tool for automated alignment trimming in large-scale phylogenetic analyses. Bioinformatics. 2009;25:1972-3.
36. Minh BQ, Schmidt HA, Chernomor O, Schrempf D, Woodhams MD, von Haeseler A, et al. IQ-TREE 2: new models and efficient methods for phylogenetic inference in the genomic era. Mol Biol Evol. 2020;37:1530-4.
37. Rusch A, Amend JP. Order-specific 16S rRNA-targeted oligonucleotide probes for (hyper) thermophilic archaea and bacteria. Extremophiles. 2004;8:357-66.
38. Stahl DA. Development and application of nucleic acid probes in bacterial systematics. In: Stackebrandt E, Goodfellow, M, editor. Nucleic acid techniques in bacterial systematics. Chichester, UK: John Wiley & Sons Ltd; 1991. p. 205-49.
39. Pernthaler A, Pernthaler J, Amann R. Fluorescence in situ hybridization and catalyzed reporter deposition for the identification of marine bacteria. Appl Environ Microbiol. 2002;68:3094-101.
40. Schaible GA, Kohtz AJ, Cliff J, Hatzenpichler R. Correlative SIP-FISH-Raman-SEM-NanoSIMS links identity, morphology, biochemistry, and physiology of environmental microbes. ISME Commun. 2022;2:52.
41. Vaughan RG, Heasler HP, Jaworowski C, Lowenstern JB, Keszthelyi LP. Provisional maps of thermal areas in Yellowstone National Park based on satellite thermal infrared imaging and field observations. Reston, VA: U.S. Geological Survey; 2014.
